# AMRrounds: Carbapenem-resistant *Acinetobacter baumannii* in the intensive care unit—when resistance meets severity

**DOI:** 10.1093/jacamr/dlaf067

**Published:** 2025-04-30

**Authors:** Davide Mangioni, Chiara Abbruzzese, Antonio Teri, Chiara Bonaiuto, Alessandro Pecere, Claudia Alteri, Ciro Canetta, Antonio Muscatello, Gian Maria Rossolini, Annapaola Callegaro, Giacomo Grasselli, Marcello Sottocorno, Mauro Panigada, Alessandra Bandera

**Affiliations:** Infectious Diseases Unit, Foundation IRCCS Ca’ Granda Ospedale Maggiore Policlinico, Milan, Italy; Department of Anaesthesia, Critical Care and Emergency, Foundation IRCCS Ca’ Granda Ospedale Maggiore Policlinico, Milan, Italy; Microbiology and Virology Unit, Foundation IRCCS Ca’ Granda Ospedale Maggiore Policlinico, Milan, Italy; Department of Experimental and Clinical Medicine, University of Florence, Florence, Italy; Microbiology and Virology Unit, Florence Careggi University Hospital, Florence, Italy; Hospital Pharmacy, Foundation IRCCS Ca’ Granda Ospedale Maggiore Policlinico, Milan, Italy; Microbiology and Virology Unit, Foundation IRCCS Ca’ Granda Ospedale Maggiore Policlinico, Milan, Italy; Department of Oncology and Hemato-Oncology, University of Milan, Milan, Italy; Acute Medical Unit, Fondazione IRCCS Ca’ Granda Ospedale Maggiore Policlinico, Milan, Italy; Infectious Diseases Unit, Foundation IRCCS Ca’ Granda Ospedale Maggiore Policlinico, Milan, Italy; Department of Experimental and Clinical Medicine, University of Florence, Florence, Italy; Microbiology and Virology Unit, Florence Careggi University Hospital, Florence, Italy; Microbiology and Virology Unit, Foundation IRCCS Ca’ Granda Ospedale Maggiore Policlinico, Milan, Italy; Department of Anaesthesia, Critical Care and Emergency, Foundation IRCCS Ca’ Granda Ospedale Maggiore Policlinico, Milan, Italy; Department of Pathophysiology and Transplantation, University of Milan, Milan, Italy; Hospital Pharmacy, Foundation IRCCS Ca’ Granda Ospedale Maggiore Policlinico, Milan, Italy; Department of Anaesthesia, Critical Care and Emergency, Foundation IRCCS Ca’ Granda Ospedale Maggiore Policlinico, Milan, Italy; Infectious Diseases Unit, Foundation IRCCS Ca’ Granda Ospedale Maggiore Policlinico, Milan, Italy; Department of Pathophysiology and Transplantation, University of Milan, Milan, Italy

## Case

**Figure 1.**
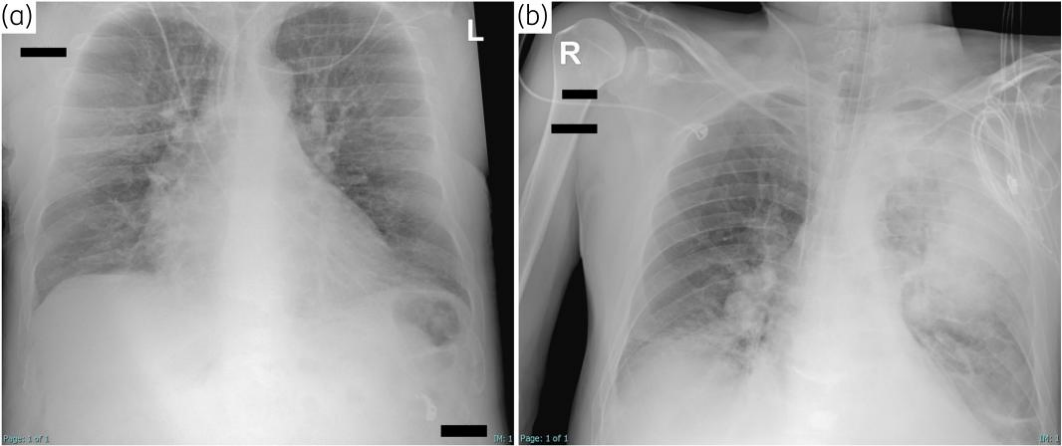
Chest X-ray performed at hospital admission (a) (no pathological findings) and 7 days later at the onset of respiratory failure and septic shock (b), which shows extensive lung consolidations in the lower right lobe and in the upper and lower left lobes.

A 65-year-old male with asthma and bronchiectasis was admitted to our hospital for mild respiratory exacerbation. At the time, the ward of admittance was handling a carbapenem-resistant *Acinetobacter baumannii* (CRAB) outbreak, and the patient underwent collection of surveillance swabs upon admission, with negative results. After complete resolution of initial symptoms, clinical conditions suddenly deteriorated 7 days from hospital admittance with fever and acute respiratory failure. Blood cultures were taken, and chest X-ray confirmed the suspicion of hospital-acquired pneumonia (Figure 1). The patient was transferred to the ICU for septic shock and acute respiratory distress syndrome (ARDS), and placed on invasive mechanical ventilation. Diagnostic bronchoscopy with bronchoalveolar lavage (BAL) was performed.

Molecular analysis of BAL (BIOFIRE^®^ FILMARRAY^®^ Pneumonia plus Panel, bioMérieux) and positive blood cultures (BIOFIRE^®^ Blood Culture Identification 2 Panel, bioMérieux) detected *Acinetobacter baumannii/calcoaceticus* complex. The patient was started on cefiderocol 2 g IV every 8 h (infused over 3 h) and ampicillin/sulbactam 9 g IV every 8 h (infused over 4 h). Amikacin (25 mg/kg IV per day) was added for broad-spectrum coverage and septic shock. Despite optimization of mechanical ventilation settings, the patient’s respiratory status continued to deteriorate, leading to life-threatening respiratory acidosis requiring veno-venous extracorporeal membrane oxygenation (VV-ECMO) as a rescue intervention.

**Figure 2.**
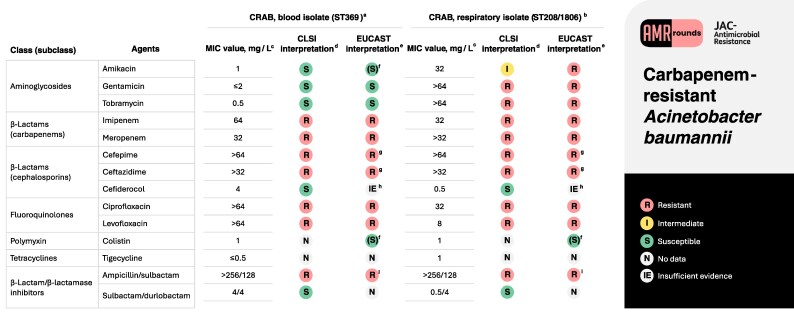
Antibiotic susceptibility profile of the isolates corresponding to the clinical case. ^a^Relevant resistome from WGS analysis for the ST369 isolate: *bla*_OXA-66_ (up-regulated by upstream insertion of IS*Aba1*), *bla*_ADC-30_ (up-regulated by upstream insertion of IS*Aba1*), *bla*_OXA-23_, *ftsI*_N392T_, Δ*pirA*, *parC*_S84L_, *gyrA*_S81L_. Resistome analysis was carried out using the ResFinder (https://github.com/cadms/resfinder), NCBI AMRFinderPlus (https://github.com/ncbi/amr) and Comprehensive Antibiotic Resistance Database (CARD) (https://card.mcmaster.ca/) platforms. The presence of IS elements in the 500 bp region upstream of the resistance genes was determined using the ISfinder tool (https://www-is.biotoul.fr/). Variant calling was performed using snippy (https://github.com/tseemann/snippy) using CP091345 as reference. Only resistance mechanisms deemed to be potentially relevant to the observed resistance phenotypes are indicated. ^b^Relevant resistome from WGS analysis: *bla*_OXA-66_, *bla*_ADC-73_ (up-regulated by upstream insertion of IS*Aba1*), *bla*_OXA-23_, *bla*_TEM-1_, *ftsI*_A515V_, *armA*, *parC*_S84L_, *gyrA*_S81L_. Resistome analysis was carried out as described in footnote a. Only resistance mechanisms deemed to be potentially relevant to the observed resistance phenotypes are indicated. ^c^Determined by broth microdilution, using iron-depleted medium for cefiderocol testing (EUCAST 15.0, 2025). ^d^According to CLSI standard M100, 35th Edition (2025). ^e^According to EUCAST 15.0 clinical breakpoints (2025). ^f^According to EUCAST 15.0 clinical breakpoints (2025), the MIC value indicates the absence of phenotypically detectable resistance mechanisms. In this case, EUCAST warns against using this agent as monotherapy due to lack of clinical evidence, while the agent could be used in combination with other effective measures. ^g^According to EUCAST guidance on ‘When there are no breakpoints in breakpoint tables?’ (September 2024), when there is a ‘dash’ instead of numerical values the microbe can be reported as resistant without further testing. ^h^According to EUCAST 15.0 clinical breakpoints (2025), the *in vitro* activity of cefiderocol for *Acinetobacter* spp. is comparable to the activity of the agent for Enterobacterales and there are also animal data to suggest efficacy. However, there are insufficient clinical data to determine a clinical breakpoint. Isolates with MIC values of ≤0.5 mg/L are mostly devoid of resistance mechanisms. Isolates with MICs of 1–2 mg/L have acquired resistance mechanisms that may result in impaired clinical response. Isolates with MIC values of >2 mg/L will likely be resistant. ^i^According to EUCAST guidance on ‘When there are no breakpoints in breakpoint tables?’ (September 2024), for the treatment of Gram-negative aerobic bacteria the clinical use of ampicillin/sulbactam should be discouraged when MIC values are higher than 8 mg/L.

On Day 4, antimicrobial susceptibility testing (AST) showed CRAB isolates from blood and the respiratory tract with different phenotypic profiles (Figure 2). WGS revealed two different bacterial strains [ST369 and ST208/1806 (Oxford scheme), respectively].

Therapy was adjusted with the addition of high-dose tigecycline (200 mg IV loading dose, then 100 mg every 12 h), and sulbactam/durlobactam was requested as an imported medication from the USA. On Day 13, upon sulbactam/durlobactam arrival, amikacin, tigecycline and ampicillin/sulbactam were discontinued and sulbactam/durlobactam 1/1 g IV every 6 h (infused over 3 h) was added to cefiderocol. Within 72 h after treatment modification, fever resolved, inflammatory markers decreased, and the patient’s oxygenation improved, allowing gradual weaning and discontinuation of VV-ECMO. Antibiotic treatment was discontinued at the end of the 14 day treatment course of sulbactam/durlobactam (i.e. Day 27). On Day 31 from ICU admission, the patient was transferred to a step-down unit.

What is the most likely explanation for this resistance profile and recommendation for treatment?

## Discussion

Over recent decades, CRAB has become one of the pathogens with the highest burden of deaths attributable to antimicrobial resistance and a critical-priority target for new antibiotic development.^1,2^ Unlike other infections with MDR pathogens, treatment options for CRAB are still very narrow.^2^ Colistin, historically considered backbone therapy, is limited by poor lung penetration, common nephrotoxicity and increasing rates of non-susceptibility. Cefiderocol, one of the newest antibiotics to enter the market, did not prove to have clear superiority compared with other treatments in randomized clinical trials (RCTs) and real-world data.^3^ In this scenario, sulbactam/durlobactam is a novel β-lactam/β-lactamase inhibitor combination that offers great potential.^2,4^ Sulbactam inhibits essential PBPs 1 and 3 of *Acinetobacter* species, but its efficacy is restricted by many β-lactamases produced by current clinical isolates. Durlobactam is a powerful serine β-lactamase inhibitor, especially of class D carbapenemases of the OXA family, which are prevalent in CRAB isolates (e.g. OXA-23 and OXA-58), able to successfully restore the activity of sulbactam.^5^ Sulbactam/durlobactam received FDA approval in May 2023, but is currently not yet approved by the EMA.

*A. baumannii* has several chromosomally encoded resistance mechanisms (e.g. multidrug efflux systems, class C and class D β-lactamases) contributing to the intrinsic antibiotic resistance profile typical of WT strains, and a remarkable ability to evolve resistance to agents potentially useful for treating *Acinetobacter* infections by chromosomal mutations or horizontal acquisition of resistance genes.^6^ Both bacterial isolates described in this work were shown to be resistant to carbapenems, third- and fourth-generation cephalosporins, fluoroquinolones and sulbactam. In addition, CRAB strain ST369 showed a higher MIC of cefiderocol, while strain ST208/1806 showed higher MICs of aminoglycosides. Both strains ST369 and ST208/1806 were shown to be susceptible to the new β-lactam/β-lactamase inhibitor combination sulbactam/durlobactam according to CLSI breakpoints, albeit with different MICs (Figure 2). Genomic characterization of the two CRAB isolates detected several acquired resistance mechanisms that could contribute to the observed phenotypes. In particular: (i) resistance to carbapenems was likely contributed to by the acquired *bla*_OXA-23_ class D carbapenemases gene, and also by IS*Aba1*-mediated up-regulation of the resident *bla*_OXA-66_ class D β-lactamase gene in the ST369 isolate, or by the A515V mutation in PBP3 in the ST208/1806 isolate; (ii) the high-level MICs of expanded-spectrum cephalosporins were likely contributed to by IS*Aba1*-mediated up-regulation of the resident ADC β-lactamases observed in both isolates; (iii) the increased cefiderocol MIC observed for the ST369 isolate was likely contributed to by inactivation of the PirA siderophore receptor; (iv) resistance to fluoroquinolones in both isolates was likely contributed to by the dual GyrA and ParC mutations in the QRDR of the respective topoisomerases; (v) resistance to aminoglycosides in the ST208/1806 isolate was likely contributed to by the acquired *armA* 16S rRNA methylase gene; (vi) the high-level MICs of ampicillin/sulbactam were likely contributed to by the complex β-lactamase profiles of the two isolates. Concerning sulbactam/durlobactam, the lower MIC observed for the ST208/1806 isolate (0.5/4 mg/L) would support a minor role of the A515V mutation of PBP3 in reducing susceptibility to sulbactam/durlobactam, while the higher MIC observed for the ST369 isolate (4/4 mg/L) would support a role of the N392T mutation in PBP3 in reducing susceptibility (Figure 2).

Besides the presence of an MDR profile, interpretation of AST results in *Acinetobacter* isolates can be even more challenging due to differences between CLSI and EUCAST with some agents (e.g. expanded-spectrum cephalosporins, cefiderocol and ampicillin/sulbactam). With cefiderocol, in particular, CLSI defines resistance when MIC values are ≥16 mg/L and recommends not to interpret disc diffusion zone diameters of ≤14 mm,^7^ while EUCAST does not yet provide clinical breakpoints but suggests considering likely resistant isolates with MIC of >2 mg/L or a zone diameter of <17 mm.^8^ In the case described, despite full *in vitro* susceptibility to cefiderocol of both CRAB isolates according to CLSI (Figure 2), effective control of the infection was obtained only when sulbactam/durlobactam was added, suggesting caution in the use of CLSI breakpoints, especially when treating severe infections. A further challenge is the interpretation of sulbactam/durlobactam MICs, for which EUCAST has not yet published clinical breakpoints.

Combination therapy for the management of severe CRAB infections is recommended by international guidelines.^9,10^ This is due to the MDR profile as well as pharmacokinetics limitations and poor efficacy/toxicity ratios of available antibiotics. Pending further data, sulbactam/durlobactam is also suggested in combination with another *in vitro* active agent.^2^ In the Phase 3 RCT ATTACK, sulbactam/durlobactam met the non-inferiority endpoint of 28 day all-cause mortality compared with colistin for the treatment of CRAB infections.^4^ Of note, imipenem/cilastatin was added to both groups as background therapy for potential coinfecting pathogens and may have contributed to the observed efficacy of sulbactam/durlobactam. A subsequent microbiological analysis of the same cohort showed that the addition of imipenem decreased sulbactam/durlobactam MIC values by 2-fold or more for five of the eight sulbactam/durlobactam-non-susceptible CRAB isolates.^11^ It may well be true with cefiderocol, which has affinity mainly for PBP3 of Enterobacterales and non-fermenting bacteria.

### Conclusions

This case illustrates the importance of integrating fundamental principles of AMRrounds such as interpretative antibiogram reading and pharmacokinetics considerations into complex and life-threatening clinical scenarios.^12^ The benefit of combination therapy for severe CRAB infections when sulbactam/durlobactam MIC values are at the susceptibility breakpoint, and which antibiotic is preferable as companion drug warrant further investigation.
